# Oral antibiotic stepdown therapy for uncomplicated streptococcal bloodstream infections

**DOI:** 10.1017/ash.2025.10110

**Published:** 2025-09-11

**Authors:** Jordan M. Loomis, Norman Mang, Jessica K. Ortwine, Larry S. Brown, Bonnie C. Prokesch, Wenjing Wei

**Affiliations:** 1 Parkland Health, Department of Pharmacy, Dallas, TX, USA; 2 Parkland Health, Department of Health Systems Research, Dallas, TX, USA; 3 University of Texas Southwestern Medical Center, Division of Infectious Diseases and Geographic Medicine, Dallas, TX, USA

## Abstract

**Objective:**

This study aimed to evaluate the role of oral antibiotic stepdown therapy in patients with uncomplicated streptococcal bacteremia. *Streptococcus* species are known pathogens in bloodstream infections (BSIs). Traditionally, BSIs were managed with intravenous (IV) antibiotics; however, growing literature supports oral antibiotics in invasive infections including BSIs.

**Design:**

This was a retrospective cohort study evaluating patients with streptococcal bacteremia between September 2019 and September 2021 at an academic safety-net hospital. Clinical outcomes were compared between patients completing treatment with IV antibiotics versus an oral stepdown regimen. The primary outcome, clinical failure, was a composite of BSI recurrence and infection-related readmission.

**Patients:**

Adult patients with at least one positive blood culture for any *Streptococcus* species were included. Patients with polymicrobial BSIs or complicated bacteremia were excluded.

**Results:**

155 patients were included, 77 (49.7%) received a course of IV antibiotics and 78 (50.3%) received an oral antibiotic stepdown regimen. Clinical failure was not different between the IV and oral groups (15.6% vs. 15.4%, respectively; OR .99 [95% CI, .41 to 2.35]). No differences were observed in 30-day all-cause mortality. Patients that received oral antibiotics had a significantly shorter hospital length of stay by 6 days (6 vs 12 d, *p* < .01).

**Conclusions:**

Our results suggest that an oral stepdown regimen for uncomplicated streptococcal BSIs is associated with similar outcomes compared to IV antibiotics. Furthermore, oral antibiotics may offer reduced length of stay and avoidance of outpatient central line placement in patients with uncomplicated streptococcal BSIs.

## Introduction

Streptococci are a diverse group of bacteria that are associated with a wide spectrum of disease manifestations, ranging from community-acquired infections to invasive infections including bloodstream infections (BSIs) and endocarditis. Intravenous (IV) antibiotics have traditionally been preferred over oral (PO) antibiotics for the treatment of BSIs.^1,2^ However, in a survey of infectious disease physicians in the United States, the decision to transition patients from IV to PO therapy was highly dependent on the organism and source of infection. Specifically, <20% of respondents were comfortable switching to PO therapy for bacteremia caused by *Staphylococcus aureus* versus >80% for beta-hemolytic *Streptococcus* species and >50% for *Enterococcus* species.^3^ Most data for PO stepdown in gram-positive BSIs involves *Staphylococcus aureus* and demonstrates conflicting results.^4–6^ Current data suggests a growing interest in PO stepdown therapy in patients who achieve clinical stability and source control with more recent randomized controlled trials demonstrating non-inferiority of PO stepdown for invasive infections such as endocarditis and osteomyelitis secondary to gram-positive pathogens.^7,8^ Advantages to oral therapies include shortened hospital admissions, reduced treatment costs, and avoidance of line-related complications.^9–11^ Most *Streptococcal* spp. are susceptible to PO antibiotics but clinical evidence supporting IV to PO stepdown for bacteremia secondary to *Streptococcus* spp. remains limited.^12–15^ The purpose of this study was to compare clinical outcomes for patients treated with a PO antibiotic stepdown regimen to a full treatment course of IV antibiotics for uncomplicated streptococcal BSIs.

## Methods

### Study design

A retrospective cohort study was performed in patients treated for streptococcal BSIs from September 2019 to September 2021 at an academic teaching hospital located in Dallas, Texas. Patients ≥18 years of age were included if they had a positive blood culture for any *Streptococcus* spp. and received treatment with an active antimicrobial agent. Patients were excluded if they had a polymicrobial BSI, if the isolated organism was deemed a contaminant by the treatment team, if the patient had persistently positive blood cultures despite appropriate IV antibiotic therapy, or if the patient was treated for a complicated BSI, defined as a presence of endovascular or metastatic focus of infection, intravascular prosthetic device, undrained abscess >5 cm, deep tissue or bone and joint involvement, extensive burn injury, infection involving the central nervous system, or death during hospitalization. In addition, patients who received ≥16 days of antibiotic therapy were excluded on the assumption that infections requiring longer than 2 weeks (± 2 d) were less likely to be uncomplicated. Patients were also excluded if they did not complete their intended antibiotic therapy due to withdrawal of care or leaving against medical advice.

Patients were grouped by individuals that received an entire treatment course of IV antibiotics and individuals that transitioned to PO antibiotics after an initial course of IV antibiotics. Manual chart review of the electronic medical record was used to confirm patient eligibility, baseline characteristics, treatment information, and clinical outcomes. Study approval was obtained by the UT Southwestern Institutional Review Board.

Patients were identified as immunocompromised if they had a congenital immunodeficiency, solid organ or bone marrow transplantation, HIV with a CD4 cell count <200 cells/mm,^3^ neutropenia (absolute neutrophil count <1000 cells/mm),^3^ and patients on immunosuppressive drugs or active chemotherapy.

### Outcomes

The primary endpoint was clinical failure, which was defined as a composite endpoint of BSI recurrence and/or infection-related readmission within 30 days of antibiotic completion.^16^ BSI recurrence was defined as a positive blood culture with the same organism identified in the index blood culture within 30 days following completion of the intended antibiotic course. Infection-related readmission was defined as readmission due to inadequate clinical improvement from the original source of infection or an adverse event related to antimicrobial therapy. Secondary endpoints included individual components of the primary outcome, hospital length of stay (LOS), and all-cause mortality at 30 days from antibiotic completion.

### Statistical analysis

Descriptive statistics were used to assess all variables including frequencies and percentages for categorical data and medians and interquartile ranges (IQR) for continuous data. Baseline characteristics, primary outcome, and other secondary outcomes used χ^2^ or Fisher’s exact test for categorical variables and Mann-Whitney U test for continuous variables. A multivariate logistic regression of the primary outcome was made using variables that had a *p* value <.10 in the univariate analysis or were found to be clinically relevant from literature review. Odds ratios (OR) with 95% confidence intervals (CI) were used to summarize logistic regression results. A two-tailed *p* value of less than .05 was considered statistically significant.

## Results

**Figure 1.**
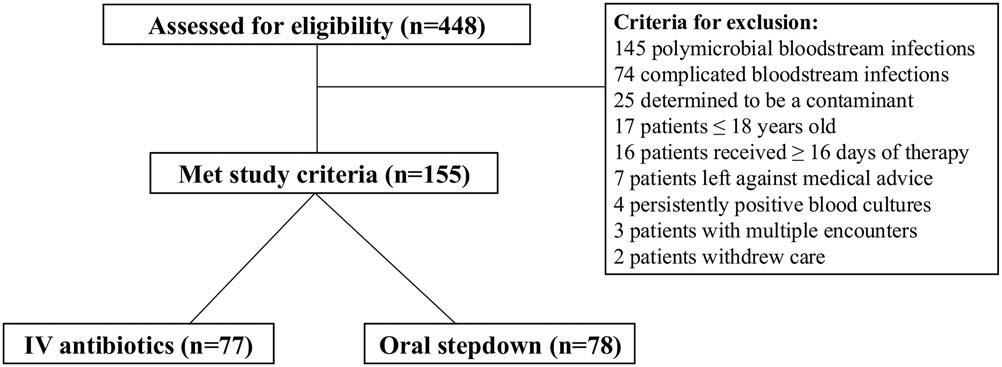
Study inclusion and exculsion.

448 patients with streptococcal bacteremia between September 2019 and September 2021 were screened for study inclusion and 155 (IV = 77; PO stepdown = 78) met eligibility for inclusion (Figure 1). The median patient age was 53 years (IQR, 43 to 64 yr), the majority of patients were male, 112 (72.3%), and only 10% had private commercial insurance. The baseline characteristics were similar between treatment groups, which included 16% immunocompromised patients (Table 1). More patients in the IV group had cirrhosis, but this was not statistically significant, (20 [26.0%] vs 11 [14.1%]; *p* = .07).

**Table 1.** Patient characteristics

Variable	IV (*n* = 77)	PO stepdown (*n* = 78)	*p* value
Age, y, median (IQR)	53 (43, 64)	52 (43, 62)	.93
Female sex, no. (%)	18 (23.4)	25 (32.1)	.23
Race/Ethnicity, no. (%)			.27
White non-Hispanic	12 (15.6)	22 (28.2)	
Black non-Hispanic	18 (23.4)	18 (23.1)	
Hispanic	44 (57.1)	36 (46.2)	
Other	3 (3.9)	2 (2.6)	
Payor Status, no. (%)			.83
Charity	42 (54.5)	38 (48.7)	
Medicaid	18 (23.4)	22 (28.2)	
Medicare	10 (13.0)	9 (11.5)	
Private	7 (9.1)	9 (11.5)	
Comorbidities, no. (%)			
Cirrhosis	20 (26.0)	11 (14.1)	.07
Diabetes	31 (40.3)	35 (44.9)	.56
Recent intra-abdominal surgery	3 (3.9)	4 (5.1)	1.00
Immunocompromised	12 (15.6)	13 (16.7)	.86
SARS-CoV-2 coinfection	6 (7.8)	4 (5.1)	.53
Empiric IV Antibiotic Therapy, no. (%)			.38
Beta-lactam	19 (24.7)	13 (16.7)	
Vancomycin	5 (6.5)	8 (10.3)	
Beta-lactam + Vancomycin	53 (68.8)	57 (73.1)	
Source, no. (%)			.03
Skin and soft tissue	26 (33.8)	44 (56.4)	
Line-related	4 (5.2)	0 (0)	
Intra-abdominal	20 (26.0)	8 (10.3)	
Respiratory	5 (6.5)	4 (5.1)	
Odontogenic	8 (10.4)	7 (9.0)	
Genitourinary	4 (5.2)	4 (5.1)	
Unknown	10 (13.0)	11 (14.1)	
Classification, no. (%)			
β-hemolytic streptococci	35 (45.5)	49 (62.8)	.03
α-hemolytic streptococci	37 (48.1)	26 (33.3)	.06
γ-hemolytic streptococci	5 (6.5)	3 (3.8)	.50
Organism, no. (%)			
Viridans group streptococci *S. salivarius* group *S. anginosus* group *S. mitis* group *S. sanguinis* group	37 (48.0) 5 (13.5) 15 (40.5) 10 (27.0) 7 (18.9)	24 (30.8) 3 (12.5) 9 (37.5) 8 (33.3) 4 (16.7)	.03
*S. pneumoniae*	0 (0)	2 (2.6)	.50
*S. bovis*	5 (6.5)	3 (3.8)	.50
*S. pyogenes*	7 (9.1)	9 (11.5)	.62
*S. agalactiae*	20 (26.0)	15 (19.2)	.32
*S. dysgalactiae*	8 (10.4)	25 (32.1)	<.01

Note: IV, intravenous; PO, oral; IQR, interquartile range.

The majority of BSIs were caused by viridans group streptococci (61 [39.4%]), followed by *S. agalactiae* (35 [22.6%]) and *S. dysgalactiae* (33 [21.3%]). The most common source of bacteremia was skin and soft tissue (70 [45.2.3%]), followed by intra-abdominal (28 [18.1%]) and odontogenic (15 [9.7%]). The source of infection remained unknown in 13.5% of cases.

**Figure 2.**
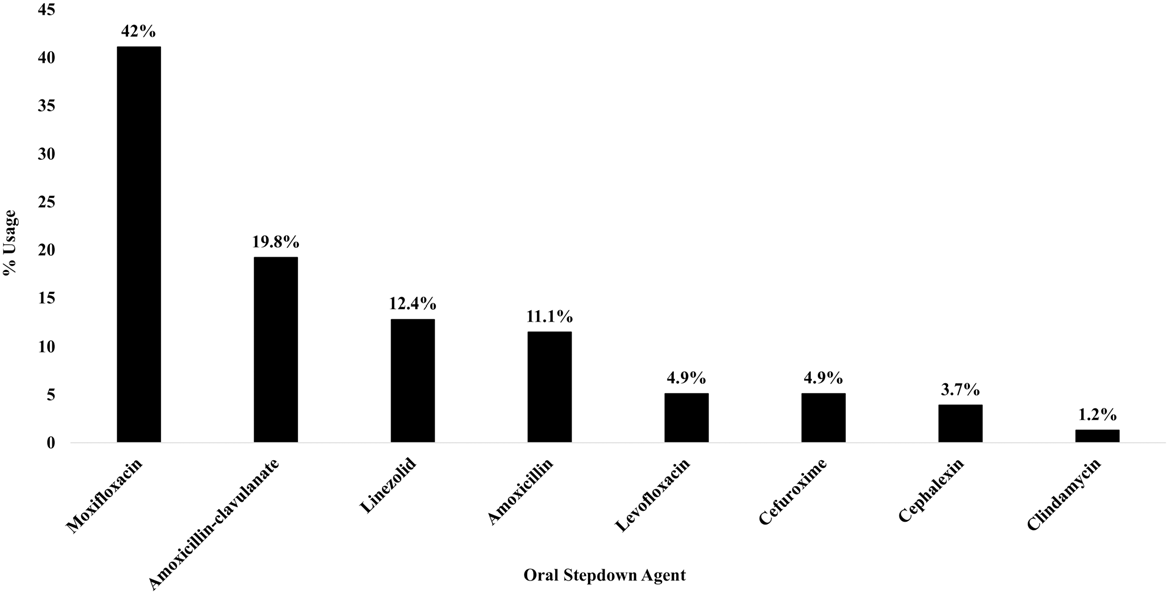
Selection of oral antibiotic stepdown agents.

All empiric IV antibiotic regimens demonstrated antimicrobial activity against the isolated organism based on predictable susceptibility or documented susceptibility reports. In patients transitioned to PO therapy, the median duration of IV lead-in therapy was 5 days (IQR, 3.5 to 7.0 d). Most patients in the IV group received targeted therapy with a beta-lactam (72 [93.5%]). An IV to PO switch was more likely for BSIs secondary to β-hemolytic streptococci (49 [62.8%]) than for α-hemolytic streptococci (26 [33.3%]). The most common PO antibiotics used for stepdown were fluroquinolones (36 [46.2%]) followed by beta-lactams (31 [39.7%]) and linezolid (10 [12.8%]) (Figure 2). The median total treatment duration in both groups was 14 days (IV group, 14 d [IQR, 8.0 to 14.0 d] vs PO group, 14 d [IQR, 14.0 to 14.5 d]; *p* < .01). Of the patients that received a full course of IV antibiotics, 33% were sent home on outpatient parenteral antimicrobial therapy.

There was no difference in clinical failure rates between IV (15.6%) and PO stepdown (15.4%) therapy (OR .99 [95% CI, .41 to 2.35]; *p* = .97). Clinical failure was primarily driven by infection-related readmission (12 [15.6%] vs 12 [15.4%]; *p* = .97), with only one patient in the IV group that had documented BSI recurrence (Table 2). Clinical worsening from the original infectious source after antibiotic completion accounted for 87.5% of infection-related readmissions. Three patients in the IV group were re-admitted for line-related complications. All-cause mortality was not different between the two groups (2 [2.6%] vs 0 [0%]; *p* = .25). Patients that received PO antibiotic stepdown therapy had a significantly shorter median hospital LOS by 6 days (12 vs 6 d; *p* < .01). The multivariate logistic regression performed on the primary outcome, clinical failure, found none of the included variables to be statistically significant (Table 3).

**Table 2.** Clinical outcomes

Outcome	IV (n = 77)	PO stepdown (n = 78)	OR (95% CI)	*p* value
Clinical failure, no. (%)	12 (15.6)	12 (15.4)	.99 (.41, 2.35)	.97
BSI recurrence, no. (%)^a^	1 (1.3)	0 (0)	…	.50
Infection-related readmission, no. (%)	12 (15.6)	12 (15.4)	.99 (.41, 2.35)	.97
All-cause mortality, no. (%)	2 (2.6)	0 (0)	…	.25
Total treatment duration, d, median (IQR)	14.0 (8.0, 14.0)	14.0 (14.0, 15.0)	…	<.01
Hospital length of stay, d, median (IQR)	12.0 (6.5, 19.5)	6.0 (4.0, 9.0)	…	<.01

Note: IV, intravenous; PO, oral; BSI, bloodstream infection; IQR, interquartile range; OR, odds ratio; CI, confidence interval. ^a^One patient with BSI recurrence included in infection-related readmission.

**Table 3.** Multivariate logistic regression for primary outcome, clinical failure

Variable	OR (95% CI)	*p* value
PO stepdown vs IV	1.20 (.47, 3.05)	.70
Age	1.00 (.96, 1.03)	.80
Cirrhosis	1.97 (.55, 6.99)	.30
*S. dysgalactiae*	.45 (.12, 1.71)	.24
β-hemolytic streptococci	1.55 (.59, 4.10)	.38

Note: IV, intravenous; PO, oral; OR, odds ratio; CI, confidence interval.

## Discussion

This retrospective cohort study of patients with uncomplicated streptococcal bacteremia found similar clinical failure rates between patients treated with IV antibiotics and those transitioned to PO antibiotics. Most failures were due to infection-related readmissions with only one case of recurrent bacteremia which occurred in the IV group. Mortality was rare and not included in our definition of clinical failure. These findings align with prior studies showing similar efficacy with PO stepdown therapy for streptococcal bacteremia, though comparisons are limited by varying definitions of uncomplicated bacteremia, clinical failure, and inclusion of variable sources, *Streptococcus* species, and therapeutic regimens. Notable differences in our patient population include more BSIs secondary to skin and soft tissue and intra-abdominal infections and lower rates of respiratory or line-related sources. Our study also showed a higher incidence of viridans group streptococcus compared to *S. pneumoniae*, which was commonly identified in prior cohorts.^17–20^ The clinical failure rate observed in the present study most closely aligns with Kang *et al*., who found no difference between PO and IV groups in re-admission rates (15% vs 20%, *p* = .4), mortality (1% vs 4%, *p* = .25), or BSI recurrence (1% vs 2%, *p* = .65).^17^ Similarly, Waked *et al*. reported no significant difference in clinical failure, a composite endpoint of 90 day readmission and/or mortality between PO and IV groups (18% vs 24 %, *p* = .23).^18^ Ramos-Otero *et al*. observed clinical failure only in the IV group (0% vs 19%, *p* = .001), primarily due to 30 day reinfection or new-onset sepsis.^19^ Lew *et al*. reported low failure rates overall, and no difference between PO and IV groups in terms of infection recurrence (0% vs 1.6%, *p* = .5), infection-related mortality (.9% vs 1.6%, *p* = 64), or infection-related readmission (4.5% vs 3.2%, *p* = .74) and low line-related complications of 3.2%.^20^ Consistent with prior studies, patients who received oral therapy in our cohort had shorter hospital stays. Kang *et al*. reported a median LOS of 5 versus 10 days, (PO vs IV, *p* < .01) and other studies similarly found LOS reductions by 4–6 days.^17–20^ In our study, patients transitioned to oral antibiotics were hospitalized six fewer days, reinforcing the established benefit of oral therapy.

Several limitations to our study are secondary to the retrospective design including susceptibility to confounding variables. A multivariate logistic regression analysis was performed and found no variables including age, cirrhosis, source, and species classification, were statistically associated with clinical failure, but sample size may have been too small to detect a difference. This cohort also did not capture post-discharge data such as medication adherence, external readmissions, or mortality events, but the patient population we serve primarily follows at our institution.

The study was not designed to provide guidance for total duration of therapy nor duration of IV therapy lead-in prior to oral transition. The average time to oral stepdown in our study was 5 days with an average cumulative duration of 14 days, which was similar to other studies.^17,19^ In light of increasing data supporting shorter durations of antibiotic therapy, future studies are needed to determine the shortest effective duration for uncomplicated streptococcal BSIs.^21^

Given significant variability amongst selected antimicrobial agents and dosing strategies, we were unable to evaluate the impact of drug selection on clinical outcomes. Fluroquinolones were more frequently prescribed than oral beta-lactams in our cohort. The ideal agent for oral stepdown therapy in streptococcal bacteremia has not been well studied. Agents such as fluoroquinolones, linezolid, and clindamycin may be favored over oral beta-lactams given higher bioavailability. However, these agents may be unnecessarily broad and have higher risks for collateral damage and adverse effects.^22^ On the other hand, *Streptococcus* spp. have low minimum inhibitory concentrations to beta-lactams; however, oral bioavailability varies amongst agents and there are limited data supporting optimal dosing and correlation to pharmacodynamic target attainment.^23^ In a study comparing oral therapies for non-staphylococcal gram-positive BSIs, the authors found no difference in clinical failure between high bioavailability agents (ie, fluroquinolones and linezolid) and low bioavailability agents (ie, beta-lactams).^14^ Similar findings were reported in a study comparing oral fluoroquinolones versus beta-lactams for uncomplicated streptococcal BSIs.^15^

In summary, our study found that in hospitalized patients with uncomplicated streptococcal BSIs, oral antibiotic stepdown therapy demonstrated similar outcomes to a continued treatment course with IV antibiotics. Important considerations prior to oral stepdown for bacteremia include clinical stability, adequate source control, and selection of an effective and highly bioavailable agent.^23–24^ Adequate investigation for need of source control and endovascular source should be evaluated for streptococcal bacteremia, especially in species with higher risk of endocarditis such as *S. gallolyticus, S. sangunis, S. gordonii, S. mitis/oralis, S. mutans, and S. cristatus*.^25^ Infectious diseases consultation has been shown to improve clinical outcomes in guiding optimal therapy as well as providing recommendations for source control.^26^ The results of this study are hypothesis-generating and may assist in stewardship efforts to reduce prolonged courses of IV antibiotics, avoid central line placement for outpatient access, and help facilitate earlier transitions of care in patients necessitating antibiotics for uncomplicated streptococcal BSIs.
